# Investigation of polyurethane pyrolysis characteristics using reactive force field molecular dynamics

**DOI:** 10.3389/fchem.2025.1691308

**Published:** 2025-12-10

**Authors:** Ting Dong, Ting Zhang, Xinghua Han, Yanhua Lan

**Affiliations:** 1 School of Chemistry and Chemical Engineering, North University of China, Taiyuan, China; 2 School of Environment and Safety Engineering, North University of China, Taiyuan, Shanxi, China

**Keywords:** polyurethane, reactive force field, product distribution, main products, pyrolysis mechanism

## Abstract

Polyurethane (PU) pyrolysis characteristics were investigated using reactive force field molecular dynamics simulations to reveal the product distribution and thermal decomposition mechanisms. A PU molecular model was constructed and simulated its pyrolysis process at 1,500–3,000 K, analyzing potential energy changes, product species, carbon-containing component distribution, main gas products, main intermediate products and initial cleavage pathways. At 1,500 K, PU mainly decomposes into NHCOO and CH_2_ fragments, with concurrent gas release. At 1,800–2,100 K, aromatic amines, olefins, and gases (including CO_2_, CO, and NH_3_) are formed through radical recombination. At higher temperatures (2,400–3,000 K), carbon rearrangement is promoted, yielding dense C_40_^+^ species alongside persistent gases. The results show that PU pyrolysis initiates with the C-O-C bond cleavage of the NHCOOCH_2_ group, generating NHCOO and CH_2_ fragments, and this cleavage occurs via a homolytic pathway. The dynamic competition between main chain scission and radical recombination drives the complex pyrolysis network, with temperature governing product diversity. This work provides microscopic insights into PU thermal degradation, supporting applications in fire safety assessment and material recycling.

## 1 Introduction

Polyurethane (PU) materials have been widely applied in traditional industries, new energy fields, and high-end fields where specific applications include furniture manufacturing, construction, transportation, and electronic devices, owing to their exceptional thermal insulation properties, lightweight characteristics, high mechanical strength, and outstanding adhesion capabilities (Hu et al., 2022; Nishiyama et al., 2020; Qin et al., 2020; Zhang et al., 2024). With the continuous increase of PU materials, the amount of waste generated has also risen significantly, posing substantial environmental threats (Yao et al., 2018). Effective management of discarded PU materials can reduce dependence on petroleum-based raw materials and mitigate the risks of environmental pollution, thus being of vital significance for the sustainable development of the chemical industry and the protection of the ecological environment (Fang et al., 2024; Oenema et al., 2022).

Within the domain of PU recycling, pyrolysis technology plays a crucial role in the resource recovery, feedstock recycling, and energy conversion of waste (Chuang, 2007). Under high temperatures, PU is decomposed into smaller molecular weight compounds, which can then be further converted into valuable chemicals or fuels (Sharuddin et al., 2016). Ha et al. studied the pyrolysis behavior of PU in a nitrogen atmosphere and noted that the pyrolysis of PU proceeds through two distinct weight loss steps (Ha and Jeon, 2024). Tang et al. investigated the dynamic pyrolysis behavior of PU and revealed that its pyrolysis yielded a diverse array of products, including alkenes, alkanes, aromatics, esters, and alcohols, while the pyrolytic gas products comprised CO_2_, CH_4_, and C_2_H_2_ (Tang et al., 2021). However, the majority of existing research in this field has centered on traditional experimental approaches, which primarily employ thermogravimetry and mass spectrometry for product analysis (Chen et al., 2023; Yao et al., 2020). The high temperature not only significantly elevates reaction complexity but also makes it challenging for traditional methods to accurately capture critical short-lived radicals and intermediate products during pyrolysis.

In contrast, the reactive force field molecular dynamics (ReaxFF MD) demonstrates its unique application advantages (van Duin et al., 2001), which can accurately simulate the bonding and debonding processes of chemical bonds at the atomic scale. ReaxFF MD has been conducted to investigate the pyrolysis and combustion processes of polymer materials (Liu et al., 2023; Wan et al., 2021; Xu et al., 2023; Yan et al., 2024; Zhang et al., 2025). Yan et al. investigated the mechanism of CO_2_ generation in HT long-flame coal molecule combustion via ReaxFF MD simulation at 2,000–3,500 K, and these simulations also demonstrate that ReaxFF MD can describe HT coal combustion behaviors (Yan et al., 2024). Zhang et al. studied the pyrolysis and combustion mechanisms in PP dust explosions through the integration of ignition experiments and ReaxFF MD simulations, which revealed the molecular-level intrinsic mechanisms of flame propagation both inside and outside the tube for PP dust (Zhang et al., 2025). Afroz et al. used ReaxFF to investigate the physicochemical, mechanical properties, stimulus response behavior of the PU rigid segment, with the stress relaxation trend consistent with experimental results (Afroz et al., 2023).

As a result, this paper will employ ReaxFF MD to study the pyrolysis process of PU at varying temperatures. The pyrolysis mechanism of PU is systematically elucidated by analyzing the potential energy of PU, the product species, the weight percentage distribution of carbon-containing species, and the composition of main gaseous products, as well as the initial cracking pathways of PU molecular chains. All of these offer microscopic insights into the pyrolysis product generation mechanisms and PU thermal degradation behaviors, which are essential for developing applications in environmental protection, material recycling, and fire safety evaluation.

## 2 Simulation details

### 2.1 Model construction

**FIGURE 1:**
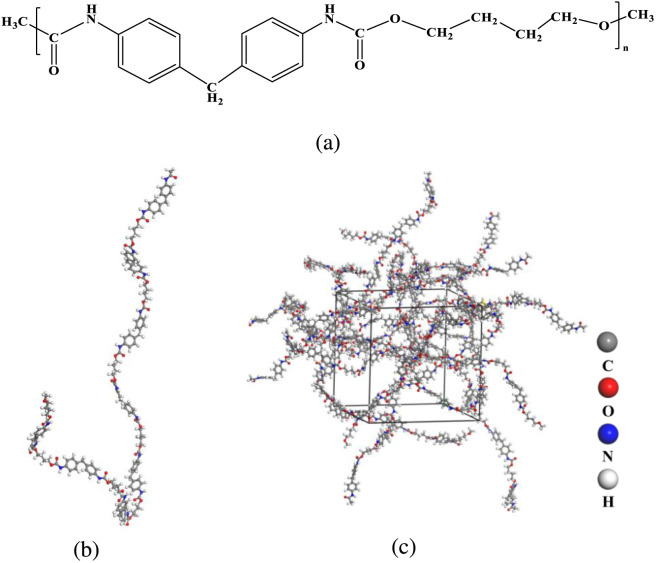
Molecular models of PU. **(a)** PU repeat unit **(b)** PU chain **(c)** PU amorphous cell with 10 chains.

The exploration of PU pyrolysis requires a suitable PU model. PU is synthesized by reacting isocyanates and polyols, and the most representative synthesis involves diphenylmethane diisocyanate (MDI) and polyols (Afroz et al., 2023; Shin et al., 2022). Based on the published article, the repeating unit of PU was first built using Materials Studio 8.0 modeling software based on the molecular structure, as depicted in Figure 1a. To generate PU polymer segments, each end of the polymer chain was terminated with a methyl (CH_3_) group (Afroz et al., 2023). To simulate the decomposition mechanism of PU, a molecular model was constructed. The value of *n* in Figure 1a is 8, and therefore the molecular formula of a single PU chain is C_154_H_166_O_32_N_16_. This chain contains 368 atoms and has a molecular weight of 2,753 g/mol (Figure 1b). The amorphous cell of PU was constructed by Rotational Isomeric State (RIS) model (Flory, 1969) to pack chains in a three-dimensional box subject to periodic boundary conditions at realistic density without close contacts. An amorphous cell containing 10 polymer chains was constructed as the initial configuration (Figure 1c), with a cubic box length of 35.70 Å and with a PU density of 1.0 g/cm^3^ (Galadari, 2023; Wei et al., 2024).

### 2.2 Molecular dynamics simulations

The PU systems were annealed from 300 to 600 K at intervals of 50 K to eliminate the local irrational structure. The condensed-phase optimized molecular potentials for atomistic simulation studies (COMPASS) forcefield (Sun, 1998) was applied to the MD simulation. The MD simulation of NPT (constant pressure and temperature) and NVT (canonical ensemble) was carried out for 500 ps with a time step of 0.1 fs, respectively. The temperature and pressure were both controlled by Berendsen method.

### 2.3 ReaxFF MD simulation

The appropriate force field is the key to molecular simulation, and the ReaxFF is the latest generation of molecular force field, which not only has the basic properties of traditional forcefields, but also can simulate the process of chemical reaction by judging the formation or breaking of chemical bonds in molecules, and iterates circularly (Li et al., 2021). After classical MD simulation, a 30 ps low-temperature equilibration simulation was performed at 300 K with the NVT ensemble by ReaxFF. The high temperature accelerated reaction kinetics was commonly adopted, in which simulations were conducted at elevated temperatures to accelerate reactions within workable computational time (Salmon et al., 2009; Sorensen and Voter, 2000). Thus six temperatures (1,500, 1,800, 2,100, 2,400, 2,700, and 3,000 K) were chosen for the products collection and analysis in the pyrolysis process of PU.

Berendsen thermostat (Arvelos et al., 2019) was used to control the temperature, and the temperature damping coefficient was set to 10 fs. The time step was 0.1 fs, and data were outputted every 50 fs. After the simulation was completed, the product distribution and main reaction mechanisms were analyzed, and the molecular structure was visually analyzed by OVITO software (Stukowski, 2009). The output file was implemented in Chemical Trajectory Analyzer (ChemTraYzer), and bond type assignment is currently implemented using the bond order perception function in Open Babel Library (Dontgen et al., 2015; O'Boyle et al., 2011).

## 3 Results and discussion

### 3.1 Potential energy and species number of the PU

The activation energy of PU decomposition was calculated. The calculated data and procedures are provided in the Supplementary Material. From Supplementary Figure S1, the *E*a of PU was 136.35 kJ/mol, which is in good agreement with the experimental data (Sun et al., 2023; Wang et al., 2014). Additionally, the linear correlation coefficient was 0.99. These results proved that the ReaxFF MD method was practicable to the study of the decomposition process of PU.

**FIGURE 2:**
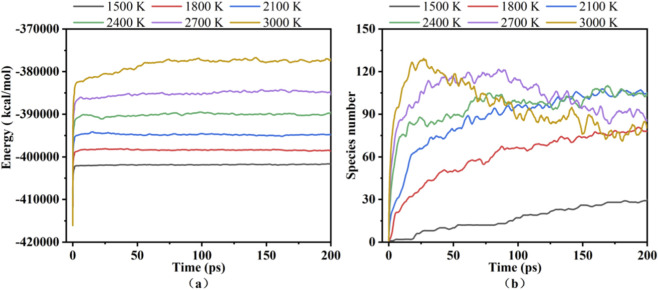
Potential energy **(a)** and species number **(b)** of the PU as a function of time at different temperatures.

The total energy of the system is composed of kinetic and potential energy, where the dynamic variations in potential energy reveal the endothermic and exothermic characteristics of the system throughout the pyrolysis process (Hong and Guo, 2017). Figure 2 displays the evolution of potential energy and species number of the PU as a function of time at different temperatures.

As shown in Figure 2a, the potential energy reflecting the endothermic energy required for bond dissociation during pyrolysis exhibits a distinct increase with temperature. When the temperature increases, the potential energy rises, and the energy absorption is enhanced. As shown in Figure 2b, at 1,500–2,400 K, the number of product species continuously increases before stabilizing. In contrast, at 2,700–3,000 K, the product species first rise sharply and then decline gradually over time. The results indicated that insufficient energy leads to incomplete reactions and fewer product species at 1,500 K. At 1,800–2,400 K, the number of product species increases with rising temperature, as higher temperatures promote collisions between intermediate products, generating a wider variety of fragment species. At temperatures of 2,700 K and 3,000 K, the number of product species exhibits a gradual decline following a peak, indicating at least two distinct chemical reaction stages during pyrolysis. PU undergoes rapid decomposition into small molecular species, with some products serving as reactants in subsequent reaction stages.

These findings are consistent with the multi-stage decomposition mechanism of PU proposed by Chen et al. (2023). In the initial two stages, C - O bond cleavage occurs via relatively weak intermolecular interactions or bond ruptures requiring moderate energy input. Conversely, the decomposition of the isocyanate segment and char formation in the final two stages involve the rupture of stronger chemical bonds within rigid isocyanate moieties, concomitantly with the formation of stable condensed-phase char structures. The latter two processes involve the absorption of more heat, thereby leading to a significant elevation in potential energy at high temperatures. These results indicate the critical role of temperature in governing pyrolysis reaction pathways and thermochemical behaviors.

### 3.2 Product distribution

**FIGURE 3:**
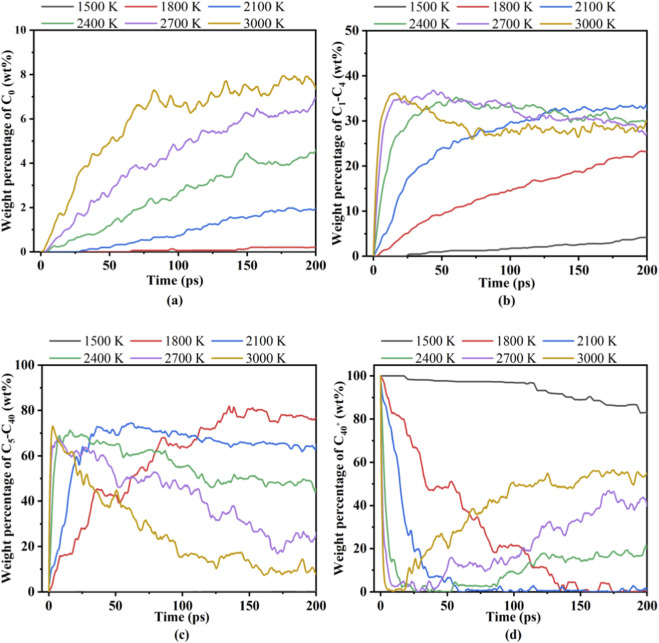
Pyrolysis composition of PU at different temperatures. **(a)** C_0_, **(b)** C_1_–C_4_, **(c)** C_5_–C_40_, and **(d)** C_40+_.

To further study the product distribution, the components with different number of carbon atoms were classified in Figure 3. Gaseous species are subdivided into C_0_ and C_1_–C_4_ according to the number of carbon atoms. The lumps of C_5_–C_40_ represent tar. C_40+_ can be considered as solid char product (Yin et al., 2018).

Figure 3 illustrates the variations in the weight percentage of C_0_, C_1_–C_4_, C_5_–C_40_, and C_40_^+^ at different temperature. At 1,500 K, the weight percentage of C_0_ and C_5_-C_40_ are negligible, C_1_–C_4_ gases are scarcely generated, and the pyrolysis products of PU are dominated by intact C_40_^+^ macromolecules. As temperature increases to 1,800–2,100 K, C_1_–C_4_ gases accumulate gradually, while C_5_–C_40_ intermediates emerge and stabilize, reflecting initial cleavage of PU chains into smaller fragments. At 2,400–3,000 K, C_1_–C_4_ gases peak rapidly within 25 ps, C_5_–C_40_ species surge initially but decline over time due to secondary reactions, and C_40_^+^ weight percentage plummets sharply in the first 10 ps. Notably, beyond 50 ps, C_40_^+^ content rises slowly with temperature, indicating recombination of C_5_–C_40_ fragments into larger organic substances. A temperature-dominated two-stage pyrolysis mechanism was revealed in this pyrolysis process.

In the moderate-temperature stage of 1,800–2,100 K, PU molecules underwent moderate decomposition to generate C_1_–C_4_ gases and C_5_–C_40_ intermediates, without complete chain scission, which was confirmed by the stabilization of C_40_^+^ content after an initial decline. When the temperature rose to 2,400–3,000 K, radical chain scission reactions dominated, producing abundant short and medium-chain fragments. Subsequently, the recombination of C_5_–C_40_ species into C_40_^+^ reflected the dynamic balance between fragmentation and recombination. This process is closely associated with the endothermic nature of PU decomposition.

### 3.3 Analysis of main products

#### 3.3.1 Main gas products

In the analysis of pyrolysis gas products of PU, the number of CO_2_ was found to be dominant, followed by CO and NH_3_, which is consistent with the conclusion by Jiao et al. (2013) pointed out that CO_2_ is the primary product at high temperatures. The number distribution of CO_2_, CO, and NH_3_ is closely linked to their formation mechanisms. As the main gas products of PU pyrolysis, the number of CO_2_, CO, and NH_3_ at different temperatures, and the production and consumption percentages of reaction paths for the CO_2_, CO, and NH_3_ at 2,700 K were statistically classified, respectively. The production and consumption percentages refer to the proportion of occurrences of this specific reaction relative to the total occurrences of the listed major reactions.

**FIGURE 4:**
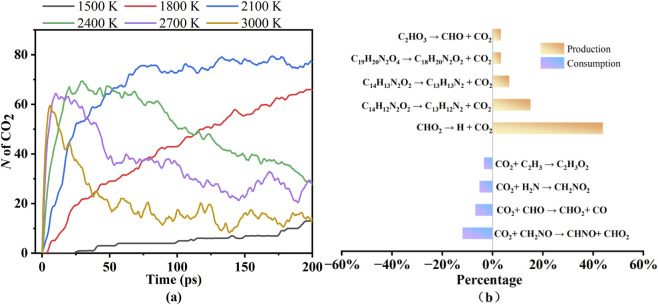
The number of CO_2_ at different temperatures **(a)** and the reaction pathways of CO_2_ at 2,700 K **(b)**.

Figure 4 presents the number of CO_2_ at different temperatures and the reaction pathways of CO_2_ at 2,700 K. According to Figure 4a, the number of CO_2_ increases sharply with rising temperature during the initial pyrolysis stage. When the pyrolysis temperature ranges from 1,500–1,800 K, CO_2_ molecules accumulate gradually over simulation time. At 2,100 K, CO_2_ molecules first surge abruptly and then stabilize. In the high-temperature range of 2,400–3,000 K, CO_2_ molecules rapidly increase to a peak value and subsequently decline, with the descent rate accelerating at higher temperatures. To investigate the balance between the CO_2_ production and consumption percentages, the reaction pathways of CO_2_ at 2,700 K are selected and depicted in Figure 4b.

The primary formation pathway for CO_2_ is CHO_2_ → CO_2_ + H. CO_2_ generation originates from the decomposition of -NH-COO- groups in the PU chain. This process is extremely rapid, causing CO_2_ to reach peak values quickly as pyrolysis temperature increases. Differences in CO_2_ production and consumption are observed across temperatures. At lower pyrolysis temperatures (e.g., 1,500–2,100 K), limited consumption pathways lead to a steady increase in CO_2_ molecules over time. Conversely, at higher temperatures (e.g., 2,400–3,000 K), the consumption rate exceeds the formation rate, causing CO_2_ molecules to decrease with faster declines at higher temperatures. This is likely attributed to enhanced participation of CO_2_ in subsequent chemical reactions at high temperatures, where increased radicals and reactive species promote CO_2_ conversion and consumption to form other gas products, such as CO and hydrocarbons.

**FIGURE 5:**
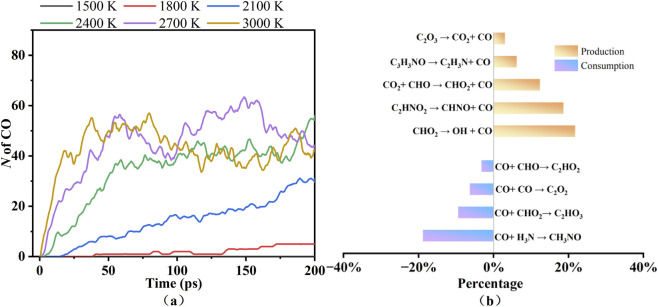
The number of CO at different temperatures **(a)** and the reaction pathways of CO at 2,700 K **(b)**.

Figure 5 displays the dynamic profiles of CO number at different temperatures and the reaction pathways at 2,700 K. As shown in Figure 5a, the number of CO gradually increases with rising pyrolysis temperature. The number of CO remains zero throughout the simulation at 1,500 K. At lower temperatures (e.g., 1,800 K, 2,100 K), the growth of the CO molecular number is much slower. By contrast, in the higher temperature range (e.g., 2,700 K, 3,000 K), the CO formation rate significantly accelerates, leading to a marked increase in molecular number. However, due to the relatively high reaction energy barriers, its total amount is significantly lower than that of CO_2_. Figure 5b reveals that the percentage of production exceeding that of consumption, resulting in a gradual increase in CO molecular number. The primary formation pathway for CO is CHO_2_ → OH + CO, while CO + H_3_N → CH_3_NO represents a key consumption pathway.

**FIGURE 6:**
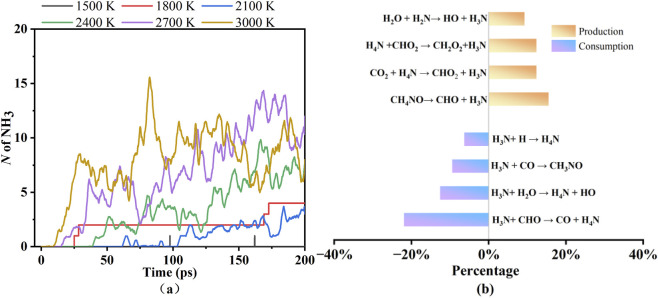
The number of NH_3_ at different temperatures **(a)** and the reaction pathways of NH_3_ at 2,700 K **(b)**.

Compared with CO_2_ and CO, the number of NH_3_ is relatively low, and it exhibits remarkable periodic fluctuations throughout the simulation time. The number of NH_3_ is constrained by the localized nitrogen enrichment in PU, as its formation requires C-N bond cleavage and radical recombination processes. Figure 6 illustrates the number of NH_3_ at different temperatures and the reaction pathways of NH_3_ at 2,700 K. The high temperature further aggravates such fluctuations, indicating that both the production and consumption of NH_3_ are characterized by high reaction rates. As shown in Figure 6a, the number of NH_3_ remain at extremely low levels, effectively approaching zero at 1,500 K and 1,800 K. Within the 2,100–3,000 K temperature, the number of NH_3_ exhibits pronounced temperature dependent oscillatory behavior over simulation time. The initial appearance time of NH_3_ progressively advances with increasing pyrolysis temperature. In Figure 6b, at 2,700 K, the production percentage of NH_3_ is slightly lower than that of consumption. Combined with the oscillatory characteristics exhibited by the time-dependent variations in NH_3_ number, this imbalance is likely attributed to the complex chemical reaction network in high-temperature environments, where competitive mechanisms exist between NH_3_ production and consumption pathways. This phenomenon indicates that high-temperature significantly enhance the kinetic rates of relevant chemical reactions, activating the NH_3_ production-consumption reaction network and leading to high-frequency, large-amplitude oscillations in its concentration.

#### 3.3.2 Main intermediate products

**FIGURE 7:**
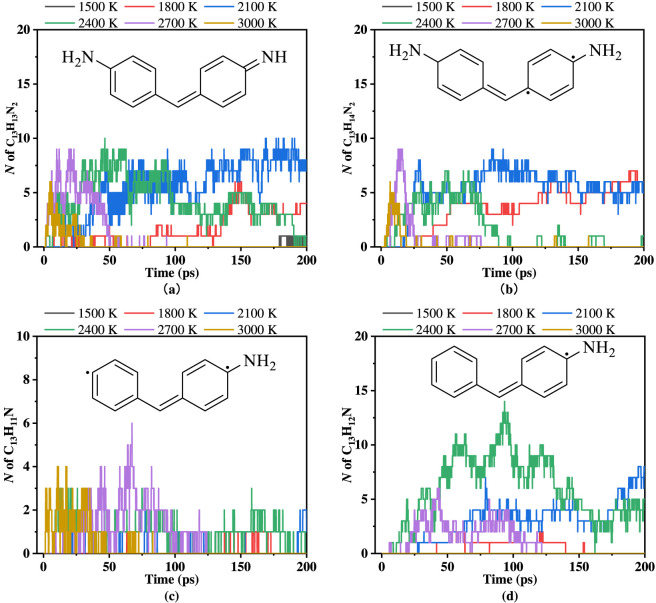
The number of C_13_H_13_N_2_ **(a)**, C_13_H_14_N_2_ **(b)**, C_13_H_11_N **(c)**, and C_13_H_12_N **(d)** at different temperatures.

Based on the analysis of the structure of PU in Figure 1 and the pyrolysis products of PU, C_13_ is a typical intermediate product. Figure 7 illustrates the molecular number of C_13_H_13_N_2_, C_13_H_14_N_2_, C_13_H_11_N, and C_13_H_12_N at different pyrolysis temperatures. As the pyrolysis temperature gradually increases from 1,500 K to 3,000 K, the emergence time of these nitrogen-containing products advances significantly, and their number initially rises. However, within the temperature range of 2,400–3,000 K, their numbers decrease, with the decline accelerating at higher temperatures. This phenomenon indicates that although intense pyrolysis reactions occur at high temperatures, excessively high temperatures lead to further cleavage or recombination of intermediate products. As shown in Figure 3, the mass fractions of C_1_–C_4_ and C_5_–C_40_ decrease with the increase in pyrolysis temperature, while the mass fraction of C_40_^+^ increases, suggesting that excessive temperature promotes the recombination of intermediate products into C_40_^+^.

**FIGURE 8:**
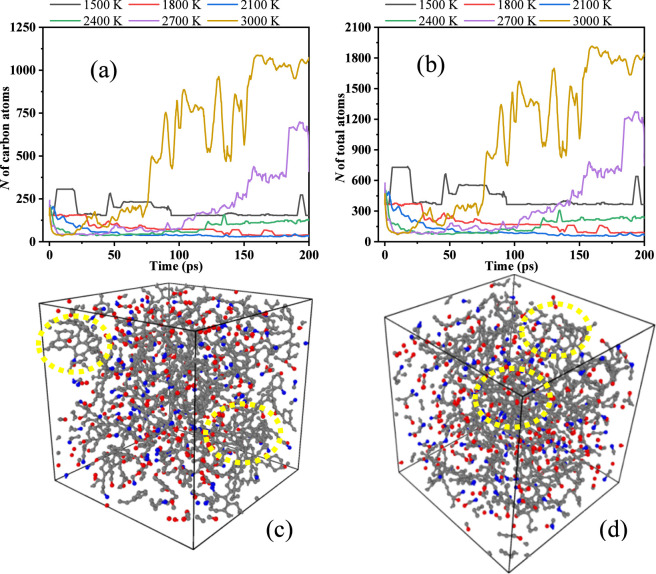
The number of carbon and total atoms in the maximum carbon fragments at different temperatures **(a,b)**, the molecular structure of PU at 200 ps (2,700 K **(c)**, 3,000 K**(d)**).

To investigate the distribution characteristics of carbon atom numbers in C_40_^+^ components, the detailed analyses of pyrolysis products at each simulation step are performed. Therein, the product with the highest carbon atom count is defined as the maximum carbon fragment. To explore the molecular structure of C_40_^+^, analysis combined with Figure 3 indicated that at pyrolysis temperatures of 2,700 K and 3,000 K, the mass fraction of C_40_^+^ exhibited a gradual upward trend during late-stage pyrolysis. OVITO software was employed to conduct visual analyses of the final products at these two temperatures. Hydrogen atoms were excluded from the analysis to enhance observation clarity. The carbon atom and the total numbers of the maximum carbon fragments and the visual structures of the final products are presented in Figure 8.

As demonstrated in Figure 8a, the maximum carbon fragment at 1,500 K contained 154 carbon atoms, which precisely matched the carbon atom number in the C_154_H_166_O_32_N_16_ fragment of PU. This finding further confirms that 1,500 K is insufficient to induce PU decomposition. With increasing temperature, the carbon number of the maximum fragment decreased abruptly. Above 1,500 K, the number remained below 154 within the range of 1,800–2,400 K. Notably, between 2,700 K and 3,000 K, the carbon number first declined rapidly and then increased, reaching approximately 1,200 atoms at 3,000 K. As shown in Figure 8b, the total number of the maximum carbon fragments and that of carbon atoms exhibit a consistent trend, with the total number reaching approximately 1,950 atoms at 3,000 K.

At 200 ps, the molecular structures of PU at 2,700 K and 3,000 K are presented in Figures 8c,d, respectively. These figures illustrate typical C_40_^+^ fragments marked with yellow circles, which exhibit benzene ring recombination structures. These observations indicate that high temperatures promote carbon recombination, leading to the formation of larger carbon fragments. At 2,700 K, the molecular structure of C_40_^+^ appeared relatively loose, characterized by sparse carbon-carbon linkages, whereas at 3,000 K, the C_40_^+^ structure became more compact with denser connections, forming a thermodynamically stable fragment. This phenomenon strongly supports the proposed dynamic competition mechanism involving main chain scission and radical recombination, highlighting that PU pyrolysis does not represent a unidirectional degradation process but rather a complex network process encompassing primary bond cleavage and secondary chain formation. These findings provide critical microscopic evidence for elucidating the distribution patterns of PU pyrolysis products and the formation mechanisms of solid residues.

### 3.4 Thermal decomposition mechanism for PU pyrolysis

**FIGURE 9:**
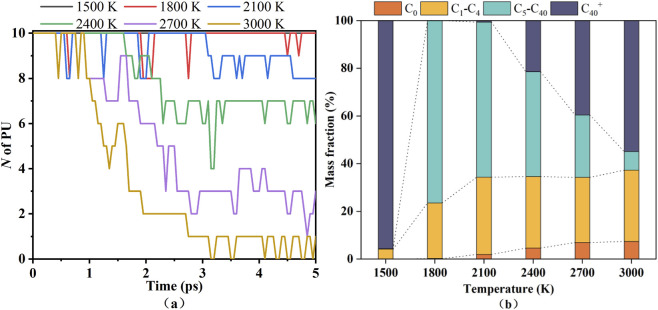
The number of PU at different temperatures **(a)** and weight percentages of C_0_, C_1_–C_4_, C_5_–C_40_, and C_40_^+^ at 200 ps **(b)**.

To deeply explore the pyrolysis mechanism of PU, simulation study on the pyrolysis process of PU within the time frame of the first 5 ps was conducted. Figure 9a depicts the number of PU across the temperature range of 1,500–3,000 K. A clear positive correlation exists between temperature and the decomposition rate of PU, with higher temperature facilitating more rapid depletion of PU molecules. Notably, within the first 3 ps, PU remains incompletely dissociated into small molecule fragments, thereby rendering this temporal regime a unique window for probing the initial pyrolysis pathways of PU.

Based on the analysis of the initially generated products, regardless of high temperature or low temperature, it can be inferred that PU pyrolysis initiates with the C-O-C bond cleavage of the NHCOOCH_2_ group, generating NHCOO and CH_2_ fragments, and this cleavage occurs via a homolytic pathway. This is consistent with the results of the stress - strain behavior of PU (Afroz et al., 2023). They pointed out that each of the chains was observed to break at the same spot on the polymer backbone where the MDI and BDO segment of the molecule were connected, more specifically, the C-O-C cleavage. None of the molecules was broken at the C-C-C or C-N-C cleavages (Afroz et al., 2023). Extracting the mass percentages of C_0_, C_1_–C_4_, C_5_–C_40_, and C_40_^+^ at 200 ps for each temperature from Figure 3 and plotting them (Figure 9b) reveals distinct evolution trends of pyrolysis fragments. At 1,500 K, where pyrolysis proceeds minimally, the C_40_^+^ macromolecular fraction dominates. With increasing temperature, the mass fraction of C_5_–C_40_ declines sharply, that of C_40_^+^ increases steadily, while C_0_ and C_1_–C_4_ exhibit negligible variations. Such trends validate the two - stage pyrolysis mechanism of PU, whereby low - temperature pyrolysis first produces gases (C_1_–C_4_) and intermediate C_5_–C_40_ fragments, which then recombine into C_40_^+^ macromolecules at higher temperatures.

**FIGURE 10:**
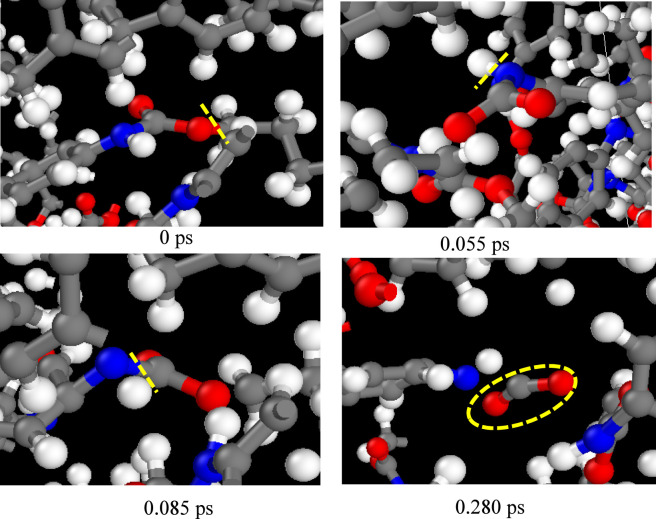
Formation of CO_2_ observed in the pyrolysis of PU.

**FIGURE 11:**
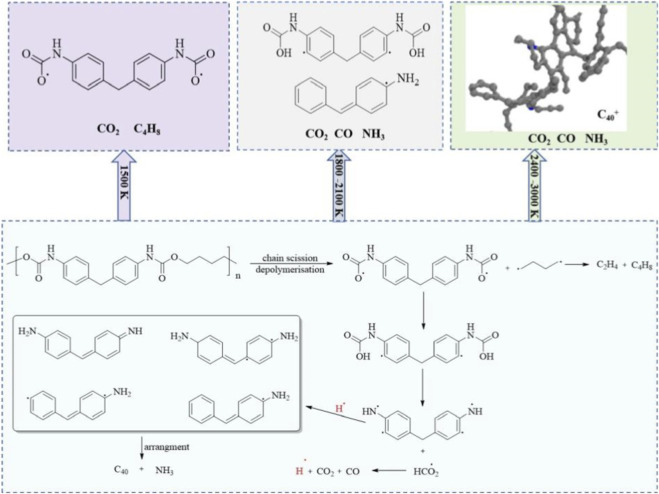
Thermal decomposition mechanism for PU pyrolysis.

The proposed CO_2_ release mechanism is presented in Figure 10. The generation of CO_2_ is detected as early as 0.28 ps, and its generation process is shown in Figure 10. The release of CO_2_ mainly results from the thermal instability of -NH-COO- in the PU chain. Under thermal conditions, these bonds preferentially cleave through hydrogen radical transfer reactions, thus releasing CO_2_. According to the types of pyrolysis products, the pyrolysis mechanism of PU was obtained, as shown in Figure 11.

Figure 11 depicts the pyrolysis mechanism of PU, initiated by C-O-C bond cleavage within the NHCOOCH_2_ moiety, yielding NHCOO and CH_2_ fragments, and this cleavage occurs via a homolytic pathway. At 1,500 K, PU remains thermally stable with intact C_40_^+^ macromolecules, characteristic of low-temperature ester scission. In the 1,800–2,100 K range, deeper decomposition generates aromatic amines, olefins, and gases (CO, NH_3_), indicative of radical recombination. At 2,400–3,000 K, high temperature induces carbon rearrangement (e.g., benzene ring recombination), forming dense C_40_^+^ networks coexisting with persistent gases.

It can be seen from Figure 11 that the temperature determines product diversity, where lower temperatures yield NHCOO and CH_2_ fragment, moderate temperatures produce aromatic amines and olefins, and high temperatures form C_40_^+^ solids, reflecting scission-recombination dynamics. The visual progression from intact PU to fragmented NHCOO and CH_2_ fragment and finally to C_40_^+^ networks maps the temperature-driven structural evolution. Gas products such as CO_2_, CO, and NH_3_ persist through all stages, while C_40_^+^ emerges at extreme temperatures (2,400–3,000 K), indicating a shift towards stable char structures.

This integration of bond cleavage, product formation, and structural evolution offers a visual roadmap for PU thermal degradation, from molecular fragmentation to solid residue formation, consistent with the mechanistic analysis presented in the study. The complexity of this pyrolysis behavior holds significant practical implications: the release of flammable C_1_–C_4_ gases requires key prevention and control in fire safety design, while the presence of tar-like C_5_–C_40_ intermediates poses challenges for PU recycling technologies. The findings further highlight the unique advantages of ReaxFF MD in capturing the complex thermochemical behaviors of PU pyrolysis, as they can reveal radical reaction pathways and dynamic equilibrium mechanisms that are difficult to observe experimentally.

## 4 Conclusion

This study employs ReaxFF MD to unravel the pyrolysis mechanisms and product distribution of PU, revealing a temperature-dependent decomposition process. At 1,500 K, PU remains thermally stable with intact C_40_^+^ macromolecules, while moderate temperatures (1,800–2,100 K) induce ester bond cleavage, yielding NHCOOCH_2_ group, generating NHCOO and CH_2_ fragments and CO_2_ alongside C_1_–C_4_ gases and C_5_–C_40_ intermediates. At 2,400–3,000 K, radical-driven scission dominates, followed by recombination of C_5_–C_40_ fragments into dense C_40_^+^ networks, demonstrating a dynamic balance between fragmentation and carbon rearrangement.

The major pyrolysis products include CO_2_, CO, NH_3_, and olefins. C_5_–C_40_ tar-like intermediates peak at moderate temperatures but decline at high temperatures due to secondary reactions, while C_40_^+^ content rebounds at 2,700–3,000 K through aromatic recombination, forming thermodynamically stable char structures. Nitrogen-containing species (e.g., C_13_H_13_N_2_) exhibit similar temperature-dependent trends, reflecting their role as transient intermediates.

Mechanistically, PU pyrolysis initiates with C-O-C bond cleavage in NHCOOCH_2_ groups, driving a complex network of chain scission and radical recombination. Temperature dictates product diversity: low temperatures favor NHCOO and CH_2_ fragment/gas release, moderate temperatures promote aromatic amine/olefin formation, and high temperatures enable solid residue generation. These findings validate ReaxFF MD as a robust tool for polymer pyrolysis studies, offering critical insights for fire safety assessment, waste recycling, and thermal material design.

## Data Availability

The original contributions presented in the study are included in the article/Supplementary Material, further inquiries can be directed to the corresponding authors.
