# Supplementary material for: Investigation of polyurethane pyrolysis characteristics using reactive force field molecular dynamics
Source: Front Chem. 2025 Dec 10;13:1691308. doi: 10.3389/fchem.2025.1691308 (PMC12745874; doi:10.3389/fchem.2025.1691308)
Supplement: Supplementary file 1 [file DataSheet1.docx]

**Investigation of polyurethane pyrolysis characteristics using reactive force field molecular dynamics**

**Dong Ting^1^, Zhang Ting^2^, Han Xinghua^1^, Lan Yanhua^2^**

^1^ School of Chemical Engineering and Technology, North University of China, 030051,Taiyuan, PR China

^2^ School of Environment and Safety Engineering, North University of China, Taiyuan, 030051, Shanxi, PR China

**1 Activation Energy**

The activation energy of PU decomposition was calculated. The calculated data and procedures are included in the supporting information. Additionally, this part of the content has been added to this manuscript. The revised content is as follows.

The Arrhenius equation can describe its quantitative law, simplistically. Taking the logarithm of both sides of the Arrhenius equation, it becomes Equation (S1).

$\ln k=\ln A-\frac{E_{a}}{RT}$ (S1)

where, *T* is the temperature, *A* is the pre-exponential factor, *E*_a_ is the activation energy and R is the ideal gas constant. Therefore, the pre-exponential factor *A* and activation energy *E*a of the pyrolysis stage can be obtained by linearly fitting the reaction rate *k* of the target model according to the relationship between ln*k* and 1000/*T*. The corresponding fitting lines and parameters are shown in Figure S1.


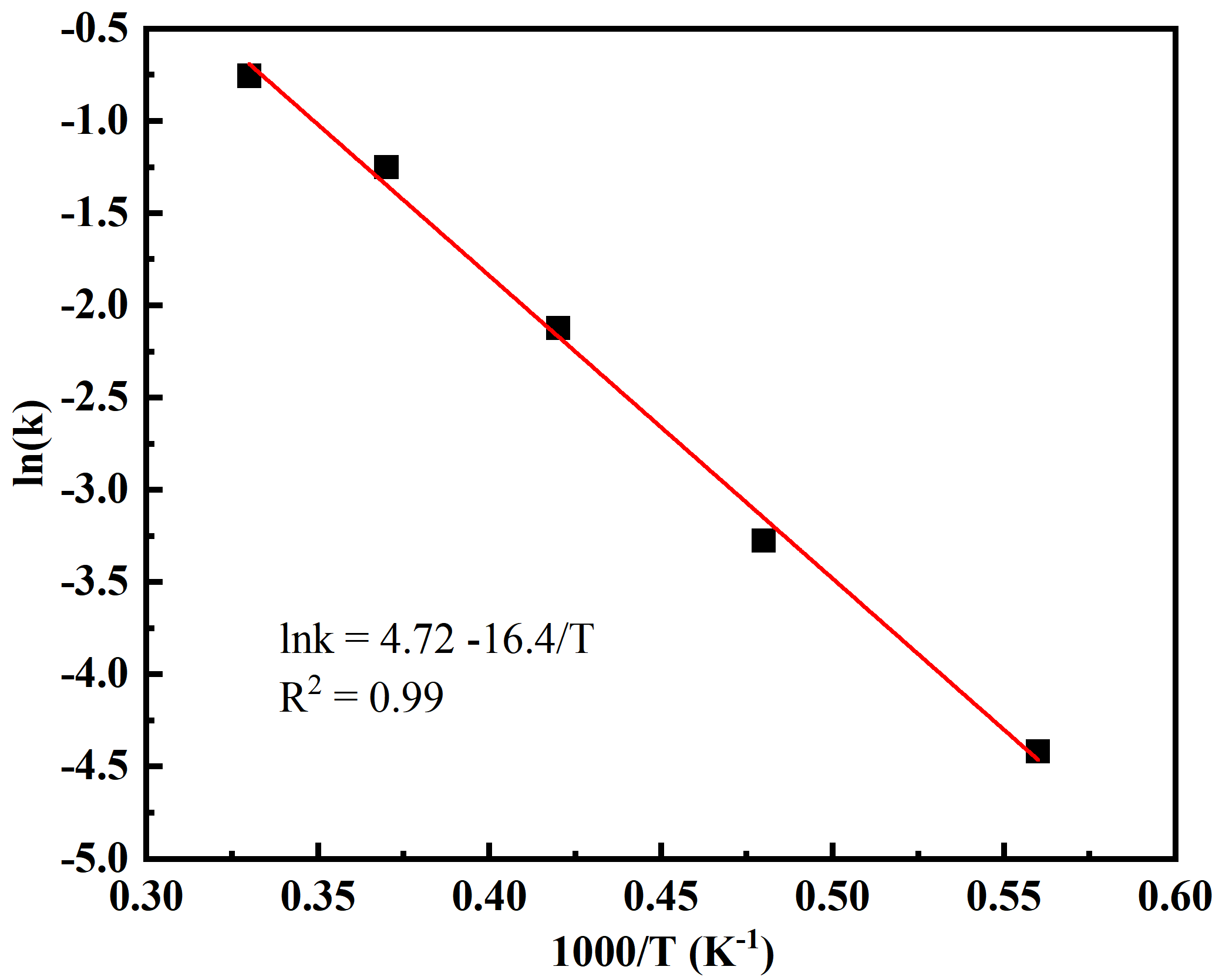


**FIGURE S1 Log fitting line of reaction rate to temperature**

From Figure R1, the *E*a of PU was 136.35 kJ/mol, which is in good agreement with the experimental data (Wang et al., 2014; Sun et al.,2023). Additionally, the linear correlation coefficient was 0.99. These results proved that the ReaxFF MD method was practicable to the study of the decomposition process of PU.

**References**

Galadari, M. (2023). Robust polyurethane-zeolite composites with diverse applications. Ph.D. Thesis, University of California, Los Angeles, CA, USA, 2023.

Wang, S., Chen, H., and Zhang, L. (2014), Thermal decomposition kinetics of rigid polyurethane foam and ignition risk by a hot particle. *J. Appl. Polym. Sci*. 131, 39359. doi: 10.1002/APP.39359

Sun, Y., Cai, D., Yang, Y., Chen, X., Wang, B., Yao, Z., et al. (2023). Investigation of the thermal conversion behavior and reaction kinetics of the pyrolysis of bio-based polyurethane: A reference study. *Biom. Bio*., 169, 106681. doi: [10.1016/j.biombioe.2022.106681](https://doi.org/10.1016/j.biombioe.2022.106681).
